# Myocardial-Treg Crosstalk: How to Tame a Wolf

**DOI:** 10.3389/fimmu.2022.914033

**Published:** 2022-05-25

**Authors:** Emil Weiß, Gustavo Campos Ramos, Murilo Delgobo

**Affiliations:** ^1^ Department of Internal Medicine I, University Hospital Würzburg, Würzburg, Germany; ^2^ Comprehensive Heart Failure Center, University Hospital Würzburg, Würzburg, Germany

**Keywords:** Tregs (regulatory T cells), Foxp3, myocardial infarction, heart, fibrosis, T-cells

## Abstract

The immune system plays a vital role in maintaining tissue integrity and organismal homeostasis. The sudden stress caused by myocardial infarction (MI) poses a significant challenge for the immune system: it must quickly substitute dead myocardial with fibrotic tissue while controlling overt inflammatory responses. In this review, we will discuss the central role of myocardial regulatory T-cells (Tregs) in orchestrating tissue repair processes and controlling local inflammation in the context of MI. We herein compile recent advances enabled by the use of transgenic mouse models with defined cardiac antigen specificity, explore whole-heart imaging techniques, outline clinical studies and summarize deep-phenotyping conducted by independent labs using single-cell transcriptomics and T-cell repertoire analysis. Furthermore, we point to multiple mechanisms and cell types targeted by Tregs in the infarcted heart, ranging from pro-fibrotic responses in mesenchymal cells to local immune modulation in myeloid and lymphoid lineages. We also discuss how both cardiac-specific and polyclonal Tregs participate in MI repair. In addition, we consider intriguing novel evidence on how the myocardial milieu takes control of potentially auto-aggressive local immune reactions by shaping myosin-specific T-cell development towards a regulatory phenotype. Finally, we examine the potential use of Treg manipulating drugs in the clinic after MI.

## Introduction

Heart and immune system development are closely intertwined, as leukocytes permeate the cardiac tissue during the embryonic stage and remain there throughout life. In addition to their housekeeping functions and recently discovered new roles (1, 2), diverse leukocyte populations are recruited by and respond to the tissue damage elicited after (MI). These responses secure the proper clearance of dying tissue, foster myocardial healing and thereby aid cardiac tissue recovery (1). However, uncontrolled long-lasting immune cell activation may also lead to cardiac damage and contribute to heart failure (HF) progression (1). Deciphering the paths and immune players responsible for proper cardiac wound healing is therefore crucial for designing new therapeutic strategies to improve post-MI recovery.

A growing body of evidence indicates that adaptive immune responses orchestrated by CD4^+^ T-cells can significantly affect myocardial repair after MI (3). While lack of CD4^+^ T-cell responses hindered myocardial healing, chronic T-cell activation can contribute to HF progression as seen in pressure overload models (4, 5). Regulatory T-cells (Tregs) have immunosuppressive and pro-healing functions that generated particular interest in understanding their role in cardiovascular diseases (6–8). A series of studies conducted in recent decades formally identified Tregs as a subset of CD4^+^ T-cells expressing the transcription factor Forkhead box P3 (FOXP3) that are involved in immunosuppression and play a vital role in maintaining immunological tolerance and overall homeostasis (9–15).

In addition to their suppressive functions, previously unknown Treg roles have recently been discovered based on their location in parenchymal rather than lymphoid tissue (16). For instance, Tregs infiltrating the visceral adipose tissue (VAT) rely on peroxisome proliferator-activated receptor (PPAR-γ) for accumulation, phenotype and function, as VAT Tregs lacking PPAR-γ cannot restore insulin sensitivity in obese mice (17). Similarly, Tregs can be recruited to the injured skeletal muscle where they mediate tissue repair *via* amphiregulin and interleukin-33 (IL-33) pathways (18, 19). Single-cell sequencing of Tregs from the skin, the colon and their respective draining lymph nodes revealed tissue-specific Treg signatures, which are present in both lymph nodes and tissue, suggesting that local draining tissue cues can shape Treg phenotypes (20). Therefore, understanding the phenotype and functions of myocardial Tregs, plus the manner in which the MI milieu influences Treg function, may form the basis for new cell-mediated therapies in cardiovascular diseases.

In this review, we will summarize the current knowledge on cardiac Tregs, including the mechanisms through which Tregs contribute to myocardial healing after infarction. We will also address how antigen specificity plays a role in CD4 heart-specific T-cell responses. Further, we will describe the heart-infiltrating Treg phenotype and how it is shaped by MI’s context. Finally, we will explore novel clinical strategies to manipulate Treg function after MI in patients.

## Bidirectional Communication Between Tregs and the Infarcted Myocardium

### Treg Effects in Myocardial Inflammation and Repair After Infarction

Investigations of heart-specific T-cell responses were initially confined to experimental models of autoimmune myocarditis, a pathological condition in which heart-directed T-cells cause myocardial damage (21, 22). More recently, however, mounting evidence has suggested that the most common myocardial diseases, namely MI and HF, can also activate antigen-specific T-cell responses, which in turn modulate myocardial inflammation and fibrosis (3, 23). MI is pathologically defined as myocardial cell death due to prolonged ischemia, which may be caused by atherosclerotic plaque disruption, interrupted oxygen supply or increased myocardial oxygen demand (24). MIs can be classified temporally according to clinical features and pathological appearance as acute (hours), healing (days) and healed (weeks) phases (24). From the immunological perspective, MI can be perceived as sterile tissue damage in the context of ischemia, resulting in the prompt release of damage-associated molecular patterns (DAMPs) and autoantigens.

**Figure 1:**
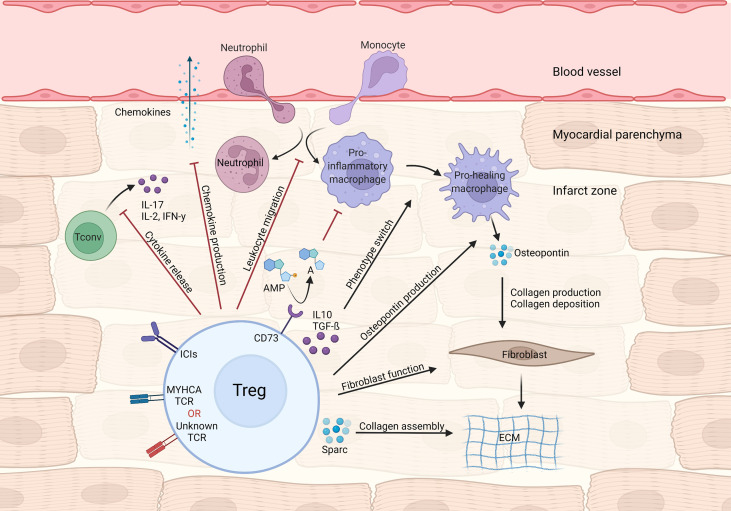
Treg-mediated effects in myocardial repair and inflammation after infarction. Tregs control local immune responses through a multitude of mechanisms, including inhibiting both canonical T_H_1/T_H_17 cytokine production and leukocyte migration. Treg cytokines (e.g. IL-10 and TGF-β) and purinergic metabolism products may modulate macrophage polarization towards a pro-healing/reparative phenotype. In addition, Tregs contribute to fibroblast activation and steady collagen deposition in the infarcted area.

During MI’s acute phase, CD4^+^ T-cells are recruited to the infarct zone and heart-draining mediastinal lymph nodes. To address the role of T-cell responses in MI outcome, Hofmann et al. applied an experimental MI model in either CD4 knockout or major histocompatibility complex class II (MHC-II) knockout mice, both of which lack functional CD4^+^T-cell responses. In all experimental settings, mice without CD4^+^ T-cell responses showed impaired myocardial leukocyte migration, reduced collagen deposition and unexpectedly decreased survival after experimental MI (25). Mice carrying a transgenic T cell receptor specific to an ovalbumin (OVA) peptide antigen (OT-II mice) also recapitulate this phenotype (25). Unlike in autoimmune myocarditis, CD4^+^ T-cell responses seen shortly after MI are mostly salutary and seem to contribute to tissue repair. In parallel to those findings, T-cells infiltrating the cardiac draining lymph nodes were shown to acquire a regulatory phenotype, that depends on TCR activation (25). Similarly, an experimental MI model in rats led to increased cardiac tissue Treg numbers, and *in vivo* Treg expansion *via* CD28 superagonistic antibody treatment resulted in improved cardiac function (26). To determine the specific contribution of Tregs to myocardial repair, Weirather et al. used gain (CD28-superagonistic antibody) and loss (FOXP3^DTR^) of function approaches in an experimental MI model (27). Tregs were found to be necessary for proper myocardial repair, as their depletion produced larger infarcts, exacerbated local inflammatory responses and hampered collagen deposition, ultimately leading to impaired survival (27). Therapeutic Treg activation favored macrophage polarization towards a pro-healing phenotype characterized by production of osteopontin, a cytokine known to potentiate collagen synthesis and deposition (27, 28). Mechanistically, canonical Treg-derived cytokines such as IL-10 and TGF-β may account for the macrophage polarization and enhanced fibrosis observed during Treg activation (29, 30). Analogous findings were observed in an ischemia-reperfusion model of MI, in which Treg depletion was associated with elevated inflammatory response, higher chemokine ligand 2 (CCL2) production and diminished fibroblast function (31). Selectively depleting Tregs in the myocardial ischemia/reperfusion model also resulted in aggravated injury, which could be rescued by transferring *in vitro* pre-activated Tregs (32). Interestingly, Tregs’ beneficial effects required intact CD39 (Ectonucleoside triphosphate diphosphohydrolase-1) signaling, suggesting that controlling purinergic metabolism may be an important Treg function in cardiovascular diseases (32). Relatedly, Borg et al. demonstrated that lack of CD73, another ectonucleotidase that converts AMP to adenosine, on CD4^+^ T-cells resulted in increased inflammatory tonus and impaired cardiac function after ischemia/reperfusion (33), and antagonizing C-X-C chemokine receptor type 4 (CXCR4) reduced scar size and attenuated cardiac remodeling after MI, through mechanisms related to augmented Treg accumulation in the infarcted region (34). Conversely, the C-C motif chemokine ligand 17 (CCL17) produced by C-C chemokine receptor type 2 (CCR2) positive monocyte-derived macrophages was shown to curtail Treg migration to the heart in a myocardial damage model induced by angiotensin II/phenylephrine treatment (35). Lack of epicardium transcription coactivators yes-associated protein 1 (YAP)/tafazzin (TAZ) signaling produced profound pericardial inflammation, fibrosis and cardiomyopathy after MI. Interestingly, knockout mice showed less Treg infiltration at the site of injury while controlled delivery of interferon gamma (IFN-γ) to the heart following MI restored Treg migration and decreased fibrosis (36), suggesting a link between YAP/TAZ and cardiac Treg function after MI. Altogether, these data suggest that CD4^+^ T-cells and, to a larger extent, Tregs favor acute myocardial healing by dampening local inflammatory responses through multiple mechanisms while fostering pro-fibrotic functions on mesenchymal cells (**Figure 1**).

Previous findings suggested that CD4^+^ T-cell activation after MI requires TCR activation *via* MHC-II (25), but the identities of those antigens and whether they are cardiac selective remained elusive. By screening a peptide library of cardiac-selective proteins that MHC-II can present, our group identified a peptide sequence spanning the cardiac-specific part of the myosin heavy alpha chain protein (MYHCA_614-629_) that drives CD4^+^ T-cell responses after MI in Balb/C mice (37–39). In adoptive transfer experiments using a transgenic TCR model against the MYHCA antigen (henceforth termed TCR-M), we showed that transferred TCR-M cells differentiated towards a regulatory phenotype in the heart, acquired a unique pro-healing gene signature in MedLNs and were associated with improved systolic function and faster collagen deposition after MI (37). In addition, heart recipients’ IL-17 production by CD4^+^ T-cells was abrogated in TCR-M-transferred mice, while recipients’ heart Treg numbers rose. Moreover, transferring *in vitro*-Treg-expanded TCR-M cells inhibited cardiac inflammatory responses (40). Remarkably, thymic epithelial cells do not express the MYHCA protein in either mice or humans; thus, central tolerance mechanisms are not functional for this antigen and peripheral presentation plays a fundamental role (41–43).

Besides myosin-specific Treg responses, polyclonal thymic-derived Tregs might also support cardiac repair after MI. In this context, Xia et al. demonstrated that thymus-derived (Helios^High^Nrp-1^High^) Tregs infiltrated the myocardium *via* the IL-33 interleukin 1 receptor-like 1(-ST2) axis and favored collagen deposition and infarct maturation through mechanisms that depend on expression of Secreted Protein Acidic And Cysteine Rich (Sparc), a gene involved in collagen calcification and extracellular matrix synthesis (44). These cells’ specificity is still largely unknown. The fact that MYHCA, the main antigen triggering peripheral Treg conversion in the heart, is not expressed in the thymus could indicate that other yet unidentified cardiac antigens might be relevant in the context of MI (**Figure 1**).

Despite the above-mentioned and well-established salutary role Tregs play during the early repair phase of MI (45, 46), it is important to stress that Tregs sometimes change their phenotype during chronic inflammatory conditions and negatively affect cardiac function by fueling pro-inflammatory mechanisms. For instance, Bansal et al. reported that Tregs lose their suppressive function and acquire features related to T_H_1 polarization in chronic ischemic HF (47). In these conditions, Tregs expressed higher TNF/TNFR1 levels and contributed to pro-fibrotic responses that led to adverse remodeling. During post-MI chronic stages, depleting Tregs using a FOXP3^DTR^ model or neutralizing anti-CD25 antibody prevented cardiac remodeling and, interestingly, reconstituted Tregs showed restored immunomodulatory activity (47), in sharp contrast to early post-MI Treg depletion (27). However, in models of chronic HF induced by stress overload, cardiac Tregs expressed high *Pdcd1* levels (encoding the inhibitory receptor PD-1), suggesting they might exert suppressive and anti-inflammatory function in this context too (48).

### How Myocardial Infarction Milieu Shapes Treg Biology

MI provides cues that may alter/shape the phenotype of local Tregs, which in turn may affect tissue repair through mechanisms beyond the immune suppression seen in autoimmunity experimental models (8, 18). Indeed, establishing a model to study cardiac-specific T-cell responses has led to important insights on how the stressed heart signals to T-cells and shapes Treg differentiation.

**Figure 2:**
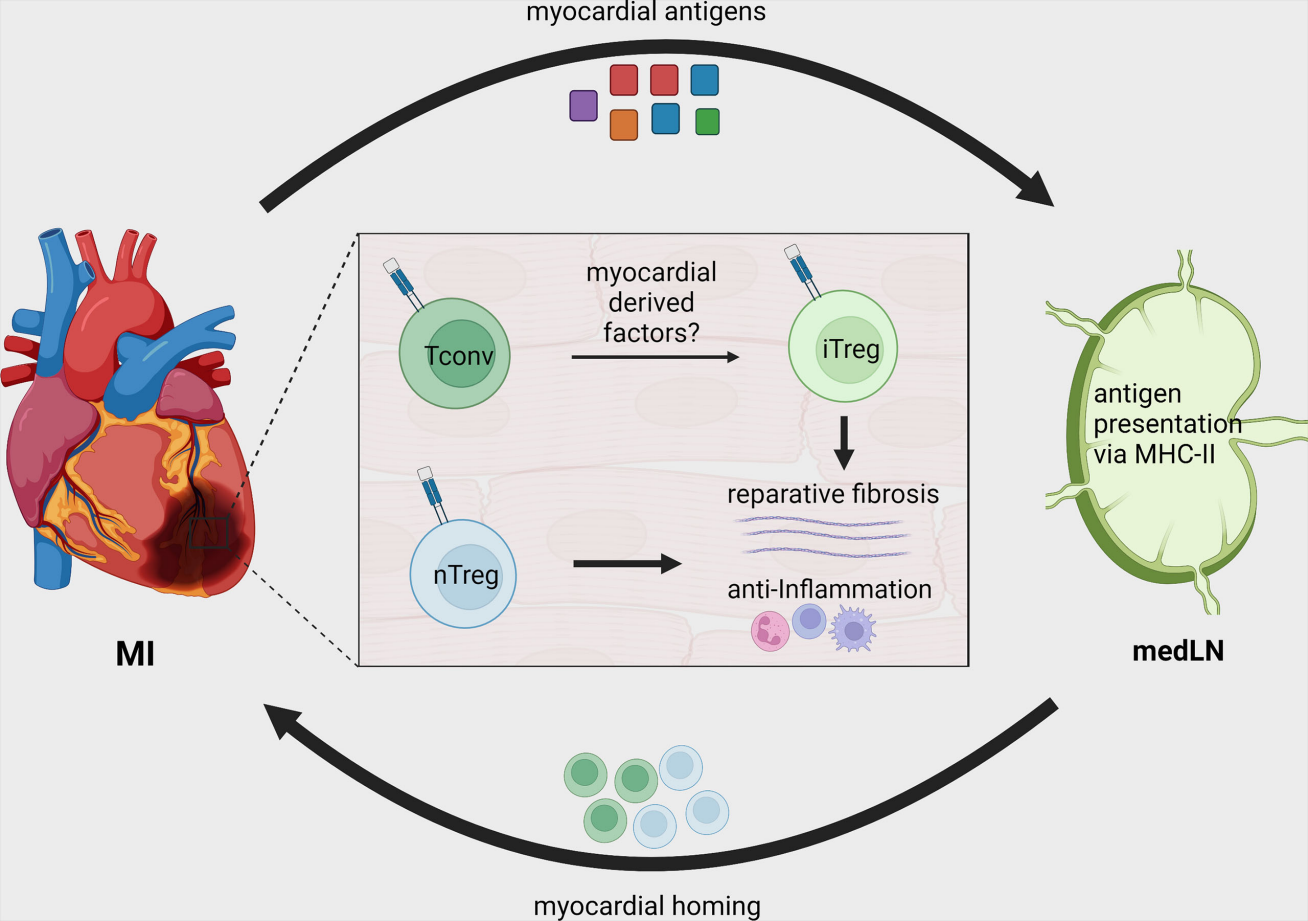
Treg recruitment and *in situ* conversion during MI. After MI, cardiac autoantigens, including MYHCA, are drained to local MedLN and presented to CD4^+^ T-cells *via* the MHC-II molecule. Naturally existing thymus derived Tregs (nTreg) and conventional T-cells are activated in the MedLN after MI and migrate to the infarcted tissue. The myocardial immune crosstalk induces myosin-specific T-cells to acquire an induced regulatory phenotype (iTreg). Both induced and naturally occurring Tregs contribute to tissue repair by modulating local inflammatory response and fostering tissue fibrosis.

The differences between baseline and cardiac TCR-M Treg frequency suggest that either the myocardium preferentially recruits Tregs or its milieu induces conventional T-cells to become Tregs. By transferring labeled conventional (CD25^-^) and regulatory (CD25^+^) TCR-M cells we showed that the myocardium attracts conventional TCR-M cells, which in turn gain FOXP3 expression, demonstrating that the infarcted heart favors *in situ* Treg conversion (37) (**Figure 2**). Strikingly, the transgenic mice bearing TCR-M cells developed spontaneous autoimmune myocarditis *via* a microbiota peptide mimicry and T_H_17 polarization, illustrating how different contexts shape the T-cell phenotypes (39). These data reveal that in infarcted tissue, cardiac-specific T-cells are poised towards an induced Treg signature that favors pro-fibrotic responses and suppresses local immune activation. The reinforcing Treg signature in myosin-specific CD4^+^ T-cells can be seen in adoptive transfer of pro-inflammatory polarized cells. For instance, *in vitro* pre-differentiated T_H_17 but not T_H_1 TCR-M cells still acquired FOXP3 expression in the heart, albeit to a lower extent than naïve TCR-M cells. Conversely, Treg-expanded TCR-M cells kept FOXP3 expression in the heart, suggesting they do not become ex-Tregs (40). More importantly, increased TCR-M Treg conversion correlated with lower inflammatory responses in the heart, regardless of infarct size, illustrating that cardiac Treg tonus directly affects local inflammatory responses (40). Taken together, these findings suggest a strong regulatory tonus imposed in the myocardium during MI’s acute healing phase affects T-helper cells that in turn module tissue inflammation. Nevertheless, these findings are yet to be confirmed in further experimental conditions and in mice with different genetic background. While the TCR-M system has been validated in Balb/C mice (MHC-II haplotype I-A^d^, I-E^d^), there are currently no cardiac antigens mapped in the widely used C57BL/6 mouse strain (MHC-II haplotype I-A^b^) or in humans. This current lack of tools to track heart-specific Tregs in C57BL/6 mice is an important limitation in the field and a major roadblock to translation. The genetic background can critically impact antigen presentation, T-cell responses, autoimmunity predisposition, myocardial function, amongst several other factors (49–52). Thus, future studies might further explore this gap and expand our toolkit to dissect myocardial T-cell responses in different mouse strains and in patients.

The myocardial injury that follows an infarction results in multiple layers of immune system activation (necrotic cell death, release of DAMPs, ischemia), yet the heart seems to tame T-cell responses and direct them toward a regulatory phenotype. Unlike tissues such as skin and liver, the adult heart possesses negligible regenerative capacity, and transient functional impairment can be life-threatening. Interestingly, the myocardium is equipped with several immune inhibitory receptors that can keep T-cell responses at bay under baseline conditions (53, 54). As a result, the heart may both prevent futile immune activation at baseline and, following injury, allow a limited response that soon shifts to a pro-resolving phase. Additionally, ligands, such as PD-L1, expressed by myocardial endothelial cells and yet undiscovered targets may contribute to *in situ* Treg polarization and inhibitory function (55, 56).

### Defining Myocardial Tregs Based on Single-Cell Transcriptomics

**Figure 3:**
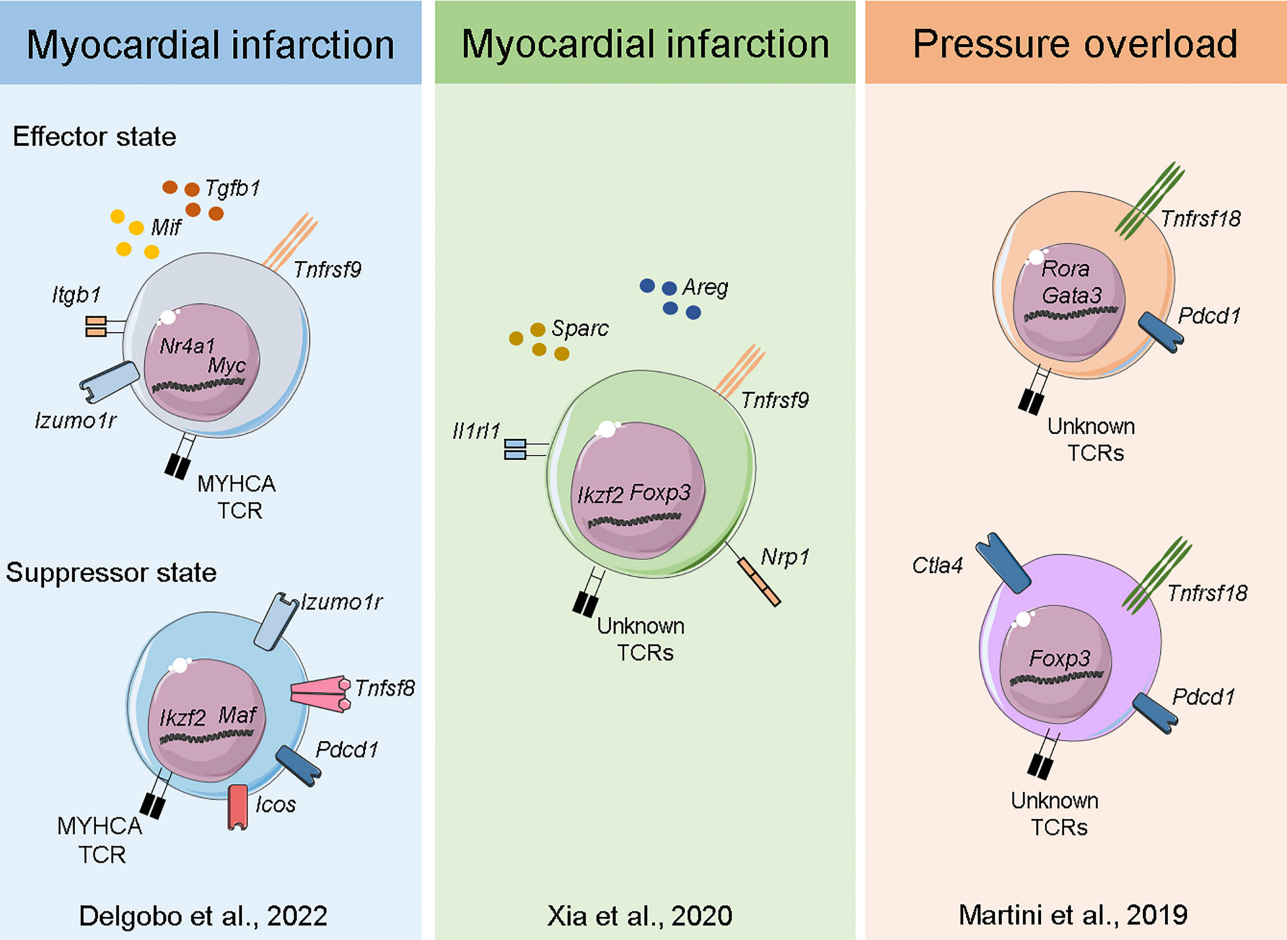
Transcriptome of myocardial Tregs in the injured heart. Single-cell sequencing of heart and MedLN T-cells after MI (40) Delgobo et al., 2022 revealed that TCR-M cells have an induced Treg signature characterized by effector-state Tregs expressing high levels of *Tgfb1* and suppressor cells expressing several immune checkpoint receptors (e.g. *Pdcd1, Icos, Tigit*). Single-cell and bulk RNA sequencing of cardiac T-cells after MI (44) Xia et al., 2020 showed Treg clonal expansion, a Treg thymus-derived signature and the production of pro-healing transcripts such as *Areg* and *Sparc*. Single-cell sequencing of myocardial leukocytes in a pressure overload model (48) Martini et al., 2019 demonstrated that two main Treg populations expanded one week post injury. Both *bona fide* Tregs and those with T_H_17/non-lymphoid gene signatures were identified, and both expressed high levels of *Pdcd1* immune checkpoint receptor.

Plasticity, polarization and adaptation are key features of the immune system, and T-cells are particularly known for adopting distinct phenotypic states dictated by the milieu. Immunological research has recently experienced a breakthrough with the advent of Single-Cell RNA sequencing (ScRNAseq) technology, which allows for unbiased, simultaneous characterization of cellular information across thousands of individual cells. Moreover, all this information can be tracked to TCRα/β sequences at the single-cell level, enabling researchers to analyze the transcriptome response, evaluate clonal expansion and eventually address expanded TCR specificity in unprecedented detail (57). Considering the complex interplay between Tregs and the injured myocardium, pioneering studies sought to resolve cardiac T-cell intricacies at the single-cell level. Xia et al. (44) combined ScRNAseq and bulk RNA sequencing to show that cardiac Tregs clonally expand and present a unique TCR repertoire. The same team also observed a transcriptome signature characterized by pro-healing genes (*Areg*), effector markers (*Tnfrsf9*) and collagen synthesis-related genes (*Sparc*). Further investigation revealed that the IL-33 axis and *Sparc* were necessary for the Treg-mediated improvement in cardiac function post MI. Martini et al. (48) used ScRNAseq to map heart leukocyte responses in a pressure-overload model. Tregs were found to be expanded after 1 week of thoracic aortic constriction (TAC) and were observed in two main clusters, one resembling *bona fide* Tregs expressing *Foxp3, Tnfrsf18* and *Ctla4* and the other expressing features of non-lymphoid and T_H_17-like Tregs such as *Rora* and *Gata3*. Remarkably, both Treg clusters expressed high levels of the checkpoint receptor *Pdcd1*, which may be associated with their suppressive function. In a mouse model of MI, our team has characterized both the endogenous (polyclonal) and myosin-specific (TCR-M cells) T-cell responses at the single-cell level in the heart and MedLNs. Intriguingly, TCR-Ms clustered separately from *bona fide* Tregs and showed a transcriptome suggesting an induced Treg signature (enriched for *Cd200, Pou2f2, Sox4* and *Izumo1r*) (40). More detailed single cell transcriptomic analyses suggested that the TCR-M cells activated after MI differentiate into two main Treg transcriptional states: a subset enriched for transcripts associated to TCR activation, cell growth/cycling and pro-fibrotic responses (*Myc*, *Tnfrsf9*, *Mif* and *Tgfb1*) and another subset expressing high levels of immune checkpoint inhibitor transcripts (*Pdcd1, Lag3, Tigit)*. However, further investigation is needed to validate these phenotypic states and eventually resolve the mechanisms underlying their differentiation and function (40) (**Figure 3**). Overall, cardiac Treg responses stem from naturally occurring and locally induced regulatory T-cells that have features linked to tissue repair, extracellular matrix organization and potent immune suppression. In addition, the molecular details of cardiac Treg priming and recruitment to the injured heart remain largely undiscovered and may explain the distinct phenotype myocardial Tregs acquire after MI.

## Scarring Versus Regeneration

Adult myocardial tissue has negligible regenerative capacity, but neonatal cardiomyocytes (CM) have a short regeneration window that lasts until shortly after birth (58, 59). Tregs may contribute to this regenerative capacity, as depleting them during pregnancy decreases fetal cardiomyocyte (CM) proliferation through paracrine mechanisms (60). In addition, neonatal cardiac regeneration is impaired in Treg-depleted mice, and Treg cell transfer to NOD/SCID mice restores their regenerative phenotype (61). Moreover, in zebrafish, which retain cardiac regenerative abilities in adulthood, disrupting Tregs dampened heart regeneration after injury. Zebrafish cardiac Tregs produce *Nrg1*, a cardiomyocyte mitogen involved in heart regeneration (62). Besides directly impacting CM proliferation, Tregs may support a pro-tolerogenic milieu in neonates while hampering pro-inflammatory T-cell activation. On the other hand, transplanting neonates with adult conventional CD3^+^ T-cells interferes with cardiac regeneration after MI, resulting in impaired function and pro-fibrotic responses, dependent on IFN-γ signaling (63). These observations regarding cardiac Tregs parallel skeletal muscle (SkM) T-cell responses after injury; in both models, Tregs constitute up to 50% of the local CD4^+^ compartment, show clonal expansion, acquire the regulatory phenotype *in situ* and are necessary for proper tissue healing (18, 19, 64). Tregs in skeletal muscle also express high levels of Helios/Nrp1 and rely on IL-33 signaling for proper tissue homing (65). While SkM Tregs enhance satellite cell myogenic activity that engenders muscle regeneration, cardiac Tregs ensure proper scar formation *via* collagen deposition in the post-mitotic myocardium (19, 26, 27, 37).

## Translational Considerations

State-of-the-art therapy for infarcted patients has significantly reduced mortality and morbidity over the years, but understanding local immune responses in the MI context may reveal immunological markers of progression toward HF and further improve patient recovery. For instance, the CANTOS (Canakinumab Anti-inflammatory Thrombosis Outcome Study) trial demonstrated that targeting pro-inflammatory cytokines in patients with previous MI lowers the rate of recurrent cardiovascular events (66).

Currently, little is known about T-cell biology in infarcted human hearts. Analyzing human cardiac autopsies showed increased Treg infiltration during the proliferative phase of MI repair. In addition, PET/CT imaging using a CXCR4 probe, as a readout for T-cell activity, revealed elevated signal in the heart draining lymph nodes of infarcted patients compared to control subjects, suggesting the existence of a similar MedLN-Heart T-cell axis following MI (37, 67). A prospective study found that higher blood Treg numbers were associated with better survival for patients with HF with reduced ejection fraction (HFrEF), indicating Tregs may influence HF progression (68). Similarly, other studies reported that infarcted patients have decreased Treg numbers in their blood and that pro-inflammatory effector T-cell expansion correlated with the occurrence of ischemic heart disease (69, 70), though other studies did not find a clear association (71). In HF patients with reduced ejection fraction, lower circulating Tregs levels correlated with higher C reactive protein and IL-6 levels and were associated with more re-hospitalizations. However, data must be interpreted carefully due the study’s small sample size (n:32) (72). The LILACS trial (Low-dose interleukin-2 in Patients with stable ischemic heart disease and acute coronary syndrome) explored the potential of Treg expansion in patients with acute coronary syndrome, in pursuit of therapeutically targeting Tregs in humans. Tregs have a high density of IL-2 receptors and are known to outcompete effector T-cells for IL-2 (73). Relatedly, low-dose IL-2 treatment induced tolerance and promoted Treg development in the context of autoimmune disease (74). In the LILACS trial, administering low-dose IL-2 (Aldesleukin) was sufficient to selectively expand Tregs but not conventional T-cells. Furthermore, the phase 1b/2a report determined the optimal IL-2 dose for Treg expansion and reported no major adverse events, thus opening the door for further studies and evaluations of the treatment’s efficacy (75, 76). Additionally, the CAR T-cell technology that has revolutionized cancer care could be used to treat cardiovascular diseases with known antigen. The work conducted by Epstein’s lab showed that CD5-targeted lipid nanoparticles carrying mRNA to reprogram lymphocytes could transiently generate CAR T-cells against fibroblast activation protein alpha (FAP) and consequently reduce fibrosis in a murine hypertensive model (77). This research opens new avenues for CAR T-cell and CAR Treg cell therapy in HF.

## Concluding Remarks

With regard to the immune system, MI substantially differs from surface/mucosal infection; MI results in abrupt release of auto-antigens and DAMPs in a sterile environment in a vital post-mitotic organ with low disease tolerance (53). Thus, optimal immune responses would restore the heart’s vital function with minimal collateral damage. We herein summarized the roles regulatory T-cells play in such processes, illustrating their protective functions during MI’s acute repair phase. In brief, this is achieved by T-cells mounting pro-tolerogenic responses to cardiac antigens and the recruitment of polyclonal Tregs to the site of injury. Further, the infarct milieu poises cardiac-specific conventional T-cells towards a regulatory phenotype. The persistent pro-inflammatory signals seen in chronic disease stages may disrupt T-cell tolerance in the myocardium. However, the molecular signals and cellular processes promoting Treg conversion and regulatory function in the injured myocardium remain largely elusive. Controlling Treg responses in myocardial diseases may lead to new therapeutic interventions aimed to restore tissue tolerance, integrity and function.

## Author Contributions

EW, GR, and MD wrote the manuscript and designed the figures. All authors contributed to the article and approved the submitted version.

## Funding

This work was supported by the Interdisciplinary Centre for Clinical Research Würzburg [E-354 to GR], the European Research Area Network—Cardiovascular Diseases [ERANET-CVD JCT2018, AIR-MI Consortium grants 01KL1902 to GR] the German Research Foundation [DFG grants 411619907 and 453989101 to GR. EW received a scholarship from the Graduate School of Life Sciences – Würzburg. GR leads projects integrated in the Collaborative Research Centre ‘Cardio-Immune interfaces’, funded by the German Research foundation (SFB1525 grant number 453989101).

## Conflict of Interest

The authors declare that the research was conducted in the absence of any commercial or financial relationships that could be construed as a potential conflict of interest.

## Publisher’s Note

All claims expressed in this article are solely those of the authors and do not necessarily represent those of their affiliated organizations, or those of the publisher, the editors and the reviewers. Any product that may be evaluated in this article, or claim that may be made by its manufacturer, is not guaranteed or endorsed by the publisher.
